# Screening of microRNA and mRNA related to secondary hair follicle morphogenesis and development and functional analysis in cashmere goats

**DOI:** 10.1007/s10142-022-00842-y

**Published:** 2022-04-29

**Authors:** Fangzheng Shang, Yu Wang, Rong Ma, Youjun Rong, Min Wang, Zhihong Wu, Erhan Hai, Jianfeng Pan, Lili Liang, Zhiying Wang, Ruijun Wang, Rui Su, Zhihong Liu, Yanhong Zhao, Zhixin Wang, Jinquan Li, Yanjun Zhang

**Affiliations:** 1 College of Animal Science, Inner Mongolia Agricultural University, Hohhot, Inner Mongolia, 010018 China; 2 College of Veterinary Medicine, Inner Mongolia Agricultural University, Hohhot, Inner Mongolia, 010018 China; 3 Key Laboratory of Mutton Sheep Genetics and Breeding, Ministry of Agriculture, Hohhot, Inner Mongolia, China; 4 Key Laboratory of Animal Genetics, Breeding and Reproduction, Inner Mongolia Autonomous Region, China; 5 Engineering Research Center for Goat Genetics and Breeding, Inner Mongolia Autonomous Region, Hohhot, China

**Keywords:** microRNA, mRNA, Cashmere goat, Secondary hair follicle, Functional analysis

## Abstract

microRNA (miRNA) is a type of endogenous short-chain non-coding RNA with regulatory function found in eukaryotes, which is involved in the regulation of a variety of cellular and biological processes. However, the research on the development of cashmere goat secondary hair follicles is still relatively scarce. In this study, small RNA libraries and mRNA libraries of 45 days, 55 days, 65 days, and 75 days of fetal skin of cashmere goats were constructed, and the constructed libraries were sequenced using Illumina Hiseq4000, and the expression profiles of miRNA and mRNA in cashmere goat fetal skin were obtained. The differentially expressed miRNAs and mRNAs in six control groups were identified and the qRT-PCR experiment shows that the sequencing results are accurate. Sixty-six miRNAs related to secondary hair follicle development were screened, and used TargetScan and miRanda to predict 33 highly expressed miRNA target genes. At the same time, 664 mRNAs related to the development of secondary hair follicles were screened, and GO enrichment and KEGG pathway analysis were performed. It was found that some miRNA target genes were consistent with the screening results of mRNAs related to secondary hair follicle development and were enriched in Notch signaling pathway, TGF-β signaling pathway. Therefore, miR-145-5p-DLL4, miR-27b-3p-DLL4, miR-30e-5p-DLL4, miR-193b-3p-TGF-β1, miR-181b-5p-NOTCH2, and miR-103-3p-NOTCH2 regulatory network related to the development of secondary hair follicles were constructed and the results of dual-luciferase reporter gene assay indicated that there is a targeted relationship between chi-miR-30e-5p and DLL4, which will provide a basis for molecular mechanism of miRNA-mRNA in the development of the hair follicles in cashmere goats.

**Supplementary Information:**

The online version contains supplementary material available at 10.1007/s10142-022-00842-y.

miRNA is a kind of endogenous non coding RNA with regulatory function found in eukaryotes. Its size is 18–25 nucleotides. Mature miRNAs are produced by a series of nuclease splicing of long primary transcripts. They recognize mRNA by base complementary pairing, and guide the silencing complex to degrade or suppress the translation of target mRNA according to different ways of base complementary pairing (Fabian et al. 2010).miRNA genes are transcribed by RNA polymerase II in the nucleus. The pri miRNA of miRNA is formed first, pri miRNA forms pre miRNA under the action of nuclease Drosha. Pre miRNA enters the cytoplasm from the nucleus under the action of specific protein, another nuclease, Dicer, cuts it to produce about 22 nucleotide lengths miRNA:miRNA double chain complex (Garzon et al. 2009).In vivo, miRNA binds to the untranslated regions (UTR) at the 3 'end of mRNA to achieve the function of inhibiting mRNA translation protein without reducing the amount of mRNA. The specific mechanism is that the seed region (seed, 2–8 nucleotide from 5'to 3' end of miRNA) and the Ago protein form complex and bind to the mRNA 3'UTR region, thus inhibiting mRNA translation (Sun et al. 2010).

There are two kinds of hair follicles in the skin of cashmere goat, namely primary hair follicle and secondary hair follicle. The primary hair follicle produces coarse hair and the secondary hair follicle produces villi. Hair follicle is a skin accessory organ with complex shape and structure. It controls the growth of hair, and its most prominent feature is regeneration. In the embryo period 45–55 days, the skin formed a complete epidermis structure, and the hair follicle has not yet occurred; in the embryo period 55–65 days, the primary hair follicles began to develop in all parts of the fetus. The keratinocytes in the basal layer of the epithelium elongated and arranged together in a palisade shape to form hair buds, but the primary hair follicles in the lateral part of the body formed later than in other parts (such as the head, shoulder and neck). In the embryo period 65 days, obvious primary hair follicle sprouts were observed in the lateral part of the body. In the embryo period 65–75 days, the primordial body of secondary hair follicles could be observed in all parts of the fetus. The secondary hair follicles began to develop and grew out of the epidermis near the primary hair follicles. Similar to the primary follicle, the secondary follicle formation in the lateral part of the body is later than that in other parts. In the embryo period 75 days, obvious secondary hair follicle sprouts were observed in the lateral part of the body (Zhang et al. 2007; Zhang et al. 2006).

The skin and hair follicle traits of cashmere goats have a direct and important impact on the yield and quality of cashmere. The ultimate goal of the research in the field of cashmere goat hair follicle growth and development is to reveal the mechanism of cashmere growth and find the important genes related to cashmere growth. The development of hair follicles may be related to some protein coding genes. At present, most of the signaling molecules that regulate hair follicle morphogenesis belong to Notch signaling pathway (Crowe et al. 1998), TGF-β signaling pathway (Ullrich and Paus 2005), Wnt signaling pathway (Beaudoin et al. 2005), FGF family (Milla 2002), etc. Some of these coding genes are stimulants and some are inhibitors of hair follicle development, which are repeatedly used and mutually regulated.

In recent years, the research on the regulation of miRNA in hair follicle development has gradually increased. Researchers use high-throughput sequencing technology to identify and predict miRNA related to skin and hair follicles. miRNA-203 is the first miRNA found to be abundantly expressed in the epidermis and hair follicles, and is also one of the miRNAs closely related to the development of skin and hair follicles (Eniko et al. 2007).Through the comparison of a large number of miRNAs, it is found that the regulatory mechanisms of miRNAs are different in the growth of feather follicles and down follicles, in the growth of feather hair follicles and mammalian hair follicles, miRNAs and their families are also different (Zhang et al. 2013).miRNA-17,miRNA-30,miRNA-15,miRNA-8 and let-7 were expressed in goat, sheep and mouse skin, and were related to skin development (Liu et al. 2010). In cashmere goats, researchers identified 22 new miRNAs and 316 conserved miRNAs in adult Inner Mongolia cashmere goats, and speculated that they may play an important role in the growth of skin and hair follicles (Liu et al. 2012); it was confirmed that miRNA-203 may regulate the development of cashmere goat follicles by targeting DDOST and NAE1 (Ma et al. 2021); 399 conserved miRNAs were identified in Shanbei white cashmere goat. Among them, 326 miRNAs were expressed in anagen, telogen, and catagem, while 3, 12, and 11 miRNAs were specifically expressed in anagen, telogen, and catagem respectively (Yuan et al. 2013); in Shanbei white cashmere goats, it was found that miRNA-206 regulates the periodic changes of hair follicles by affecting the expression of genes related to hair follicle initiation and development to a certain extent (Zhou 2016); the key genes and miRNAs affecting the proliferation and differentiation of hair follicle stem cells were screened by using biological information analysis, and the regulatory effects of miR-22-5p and let-7b-5p on the proliferation and differentiation of hair follicle stem cells were verified (Hailong 2019); through the combined analysis of mRNA and miRNA, the miRNAs related to hair follicle development in Inner Mongolia cashmere goat embryo was screened, and its function was verified (Han et al. 2020). Therefore, this study explored the molecular regulation of miRNA in the initiation and development of cashmere goat secondary hair follicles, and provided theoretical basis for further understanding the molecular regulation mechanism of cashmere growth.

## Methods

### Animals and samples

In this experiment, 3-year-old ewes were selected from Jinlai Animal Husbandry in Inner Mongolia for estrus synchronization, and the mating time was recorded. After taking skin samples from 45, 55, 65, and 75 days of gestation, they were immediately treated with DPEC water and placed in liquid nitrogen. Then it was stored in the refrigerator at—80℃ for subsequent sequencing and qRT-PCR experiments.

### RNA library construction and sequencing

In this study, 9 small RNA libraries and 9 long RNA libraries were prepared with three replicates in each group. Briefly, total RNA was isolated and purified using Trizol reagent (Invitrogen, Carlsbad, CA, USA), following the manufacturer's procedure. The amount of RNA and purity of each sample were quantified using NanoDrop ND-1000 (NanoDrop, Wilmington, DE, USA). The RNA integrity was assessed using Agilent 2100.Small RNA sequencing library preparation uses TruSeq Small RNA Sample Prep Kits (Illumina, San Diego, USA). About 5 µg of total RNA was used to deplete ribosomal RNA according to the instructions of the Ribo-Zero™ rRNA Removal Kit (Illumina, San Diego, USA), and the remaining RNA fragments were reverse transcribed using an RNA-seq Library Preparation Kit (Illumina) to form the final cDNA. Finally, we performed the paired-end sequencing on an Illumina Hiseq4000 (LC Bio, Hangzhou, Zhejiang, China), following the vendor’s recommended protocol.

#### Small RNA sequencing data analysis

ACGT101-miR (LC Sciences, Houston, Texas, USA) was used to analyze the miRNA. The analysis process of the software was as follows: clean reads were obtained from the original data after quality control, and the 3 'adapters was removed by clean reads, and the length of the 18-26nt sequence was screened. Then, the remaining sequences were aligned to various RNA databases (excluding miRNA), such as mRNA database, rfam database (including rRNA, tRNA, snRNA, snoRNA, etc.) and RepBase database (repetitive sequence database), and filtered. The final data obtained is the valid data, which can be used for subsequent small RNA data analysis.

#### Long RNA sequencing data analysis

Firstly, Cutadapt (Martin 2011) was used to remove the reads that contained adaptor contamination, low quality bases and undetermined bases. Then sequence quality was verified using FastQC (http://www.bioinformatics.babraham.ac.uk/projects/fastqc/). We used Bowtie2 and Hisat2 (Langmaed and Salzberg 2012) to map reads to the genome of goat. The mapped reads of each sample were assembled using StringTie (Pertea et al. 2015). Then, all transcriptomes from cashmere goat samples were merged to reconstruct a comprehensive transcriptome using perl scripts. After the final transcriptome was generated, StringTie (Pertea et al. 2015) and Ballgown (Frazee et al. 2015) were used to estimate the expression levels of all transcripts.

#### Differentially expressed mRNAs

StringTie (Pertea et al. 2015) was used to perform expression level for mRNAs and lncRNAs by calculating FPKM (Trapnell et al. 2010). The differentially expressed mRNAs and lncRNAs were selected with log2 (fold change) > 1 or log2(fold change) < -1 and with statistical significance (*p* value < 0.05) by R package-Ballgown (Frazee et al. 2015).

#### Differentially expressed miRNAs

Differential expression of miRNAs based on normalized deep-sequencing counts was analyzed by selectively using Fisher exact test, Chi-squared 2 × 2 test, Chi-squared nXn test, Student *t* test, or ANOVA based on the experiments design. The significance threshold was set to be 0.01 and 0.05 in each test.

#### Validation of sequencing data

Four differentially expressed miRNAs and 4 differentially expressed mRNAs were randomly selected to verify the sequencing results. The primers were designed according to the stem loop primer method of miRNA and the random primer method of mRNA. U6 and β-actin were used as miRNA and mRNA reference genes respectively. After the primers were designed, Primescript™ RT reverse transcription kit and SYBR Premix Ex Tap™ II quantitative kit were used to detect the expression of target miRNA and mRNA in the skin tissue of cashmere goats during fetal period, strictly referring to the instructions of the kit. All data were obtained by repeated experiments for three times, and the relative expression was calculated by 2 ^− ΔΔ CT^ method (Schmittgen and Livak 2008).

## Screening of miRNAs and mRNAs related to the development of secondary hair follicles

In order to screen the miRNAs and mRNAs related to the development of secondary hair follicles in cashmere goats, the (d65 vs d45, d65 vs d55 and d55 vs d45) differentially expressed miRNAs or mRNAs are combined (stage A) and (d75 vs d45, d75 vs d55, d75 vs d65) differential miRNAs or mRNAs are combined (Stage B) Then make Venn diagrams for stage A and stage B.The differentially expressed miRNA and mRNA shared by stage B and stage A are screened out, and the remaining differential miRNA and mRNA of stage B are used as miRNA and mRNA related to the development of secondary hair follicles.

### GO enrichment analysis and KEGG pathway analysis

Gene ontology analysis and KEGG pathway analysis were carried out on the screened mRNA. On one hand, they were annotated, on the other hand, in order to find out the main related functions of these candidate genes.We mapped the differentially expressed genes to Go database (http://www.geneontology.org/) and the number of genes in each term is calculated to get the gene list and gene number statistics with a go function. Go has three ontologies, which describe the molecular function, cellular component and biological process of gene respectively. At the same time, based on KEGG (http://www.genome.jp/kegg/), it is helpful to further understand the biological function of genes.

### Combined analysis of miRNA and mRNA data

According to the expression level of miRNA, we selected the important miRNA related to secondary hair follicle development and used Targetscan (http://www.targetscan.org/mamm_ 31 /) and miRanda (http://www.microrna.org/microrna/home.do) two softwares predicted the target genes of miRNA, further crossed the predicted target genes with the genes enriched in the signal pathway related to hair follicle development, and initially constructed the miRNA -mRNA regulatory network related to secondary hair follicle development.

### Dual-luciferase reporter gene assay

Primers for amplifying DLL4 and the DLL4 3’-untranslated region (UTR) were designed based on the gene sequence in GenBank, and the 3’ -UTR sequence of the gene was amplified by PCR using Cashmere goat genomic DNA as template. PCR products were cloned into the pSI-checK2 dual-luciferase reporter gene vector (Hanheng Biotechnology Co., Ltd., Shanghai, Municipality, China) to construct the psiCHECK2-DLL4-WT. The mutant psiCHECK2-DLL4-MUT construct was generated by mutating the miRNA-binding sequence to the complementary sequence using overlapping extension PCR.chi-miR-145-5p mimics and chi-miR-30e-5p mimics were synthesized by (Hanheng Biotechnology Co., Ltd.,

Shanghai, Municipality, China). The miRNA mimics were transfected into HEK 293 T cells using the LipoFiter transfection reagent according to the manufacturer’s instructions. For DLL4 luciferase assays, the HEK 293 T cells were transfected with miRNA mimics and either the psiCHECK2-DLL4-WT or mutated psiCHECK2-DLL4-Mut reporter plasmid. At 48 h post-transfection, luciferase activity was measured using a dual-luciferase reporter assay system (Promega) according to the manufacturer’s instructions. The relative luciferase activities were calculated by comparing the Firefly/Renilla luciferase activity ratio.

## Results

### Overview of the sequencing data

**Figure 1:**
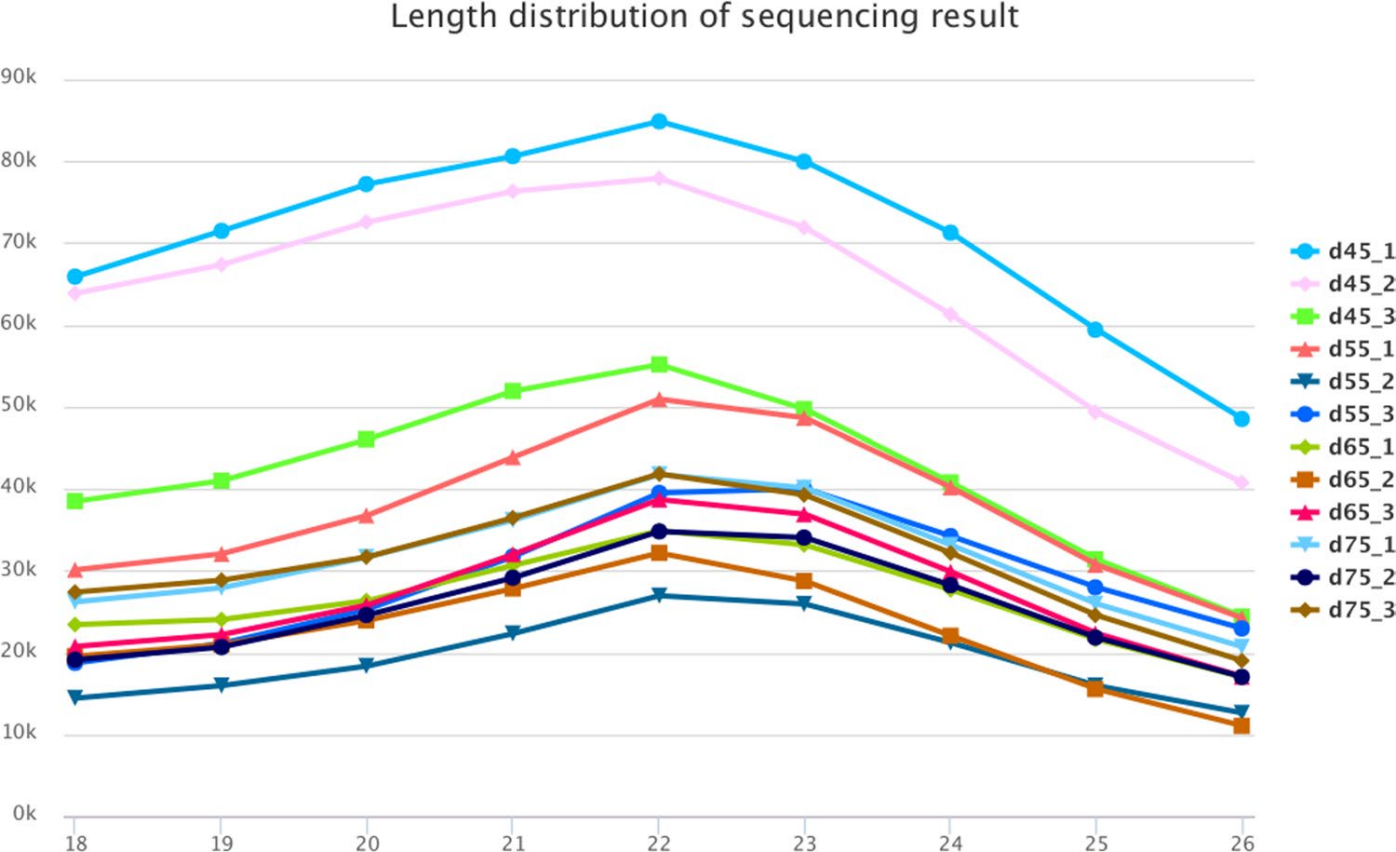
miRNA length distribution.

As a result, the small and long RNA sequencing provided by Illumina technology generated an average of 11–14 million single-end raw reads and 82–96 million paired-end raw reads from each skin sample. For the small RNA sequencing data, the sequencing error rate is about 1%, the Q20 (the proportion of bases with quality value ≥ 20, error rate < 0.001) of each library was above 99.4% and the Q30 (the proportion of bases with quality value ≥ 30, error rate < 0.001) was above 98.3%, and the GC content is more than 52% (Table 1), indicating that the small RNA sequencing results are good and can be used for subsequent analysis. Most of the miRNA sequences were distributed between 20 and 24nt, which was consistent with the typical characteristics of Dicer enzyme cleavage (Fig. 1). For the sequencing data of long RNA, the number of reads compared to the reference genome accounted for more than 94% of the valid reads. The Q20 (the proportion of bases with quality value ≥ 20, error rate < 0.001) of each library was above 99.90% and the Q30 (the proportion of bases with quality value ≥ 30, error rate < 0.001) was above 98.1% (Table 2). Therefore, the utilization rate of data is normal, and the quantity and quality of the original data can meet the requirements of subsequent analysis.

**Table 1:** Quality control statistics of small RNA data

Sample	Total Reads	Error%	Q20%	Q30%
d45_1	11,735,989	0.97	99.80	99.36
d45_2	13,989,155	0.99	99.73	99.11
d45_3	15,841,243	0.99	99.70	99.03
d55_1	20,605,620	0.99	99.71	99.09
d55_2	8,348,513	0.98	99.74	99.16
d55_3	13,703,793	0.98	99.76	99.24
d65_1	11,627,944	0.97	99.82	99.40
d65_2	10,973,757	0.97	99.80	99.36
d65_3	11,508,125	1.05	99.46	98.33
d75_1	13,305,263	0.97	99.78	99.35
d75_2	10,098,752	0.97	99.78	99.33
d75_3	15,126,878	0.96	99.82	99.42

**Table 2:** Quality control statistics of long RNA data

Sample	Raw Data	Valid Data	ValidRatio(reads)	Q20%	Q30%
	Read	Read			
d45_1	83,377,840	80,991,260	97.14	99.97	98.33
d45_2	80,073,650	77,655,516	96.98	99.97	98.33
d45_3	91,499,974	87,070,648	95.16	99.98	98.41
d55_1	100,354,248	96,199,486	95.86	99.98	98.45
d55_2	91,779,488	88,089,256	95.98	99.98	98.53
d55_3	94,888,486	91,438,886	96.36	99.98	98.48
d65_1	90,684,330	87,483,330	96.47	99.97	98.17
d65_2	102,708,880	99,047,498	96.44	99.98	98.47
d65_3	80,732,910	77,823,688	96.40	99.97	98.34
d75_1	81,140,600	77,983,860	96.11	99.98	98.45
d75_2	83,141,742	80,115,068	96.36	99.98	98.46
d75_3	82,917,418	79,990,864	96.47	99.97	98.33

### Differentially expressed miRNAs and mRNAs

**Fig. 2:**
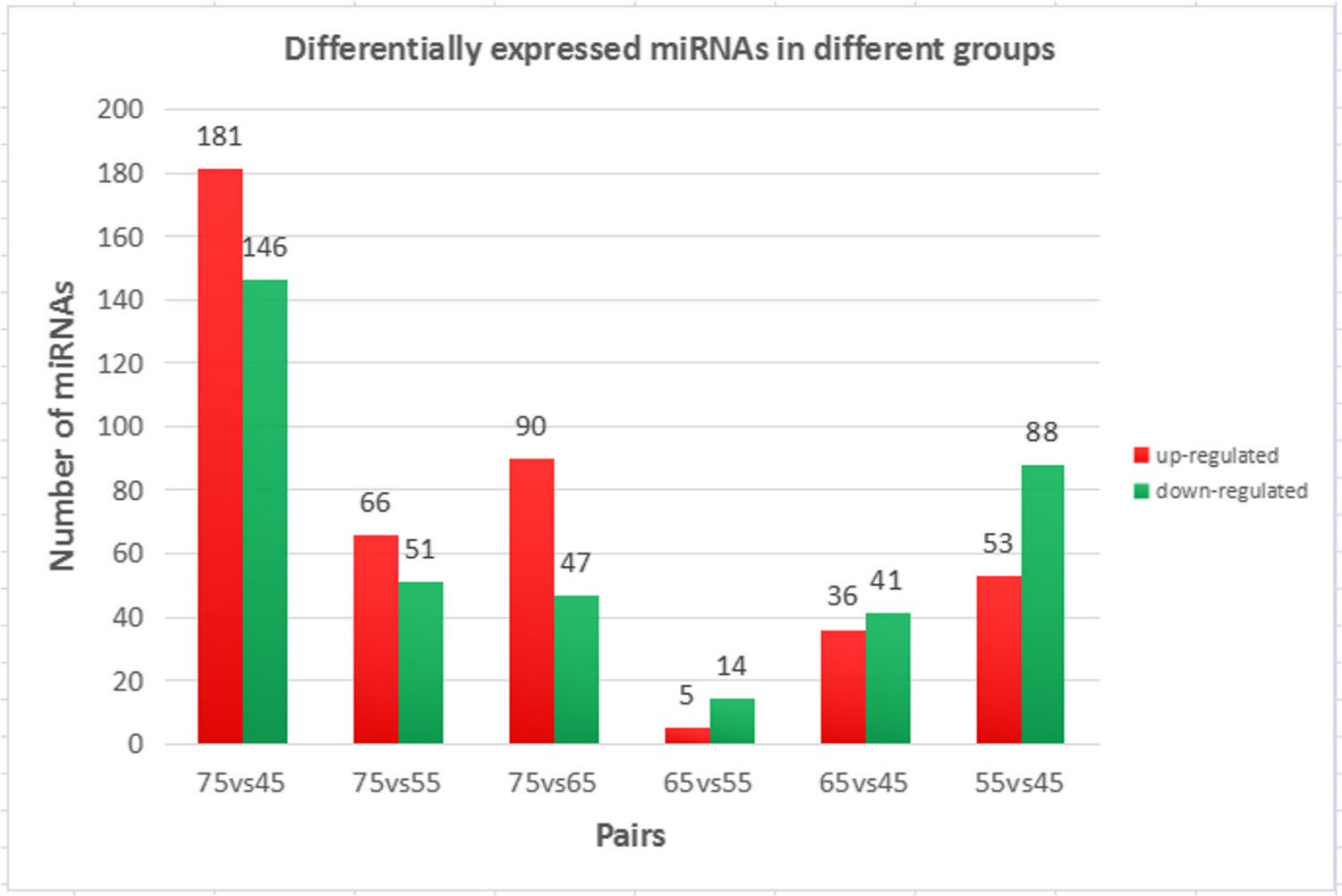
Differentially expression miRNAs in different groups. The red column represents upregulated miRNA and the green column represents downregulated

**Fig. 3:**
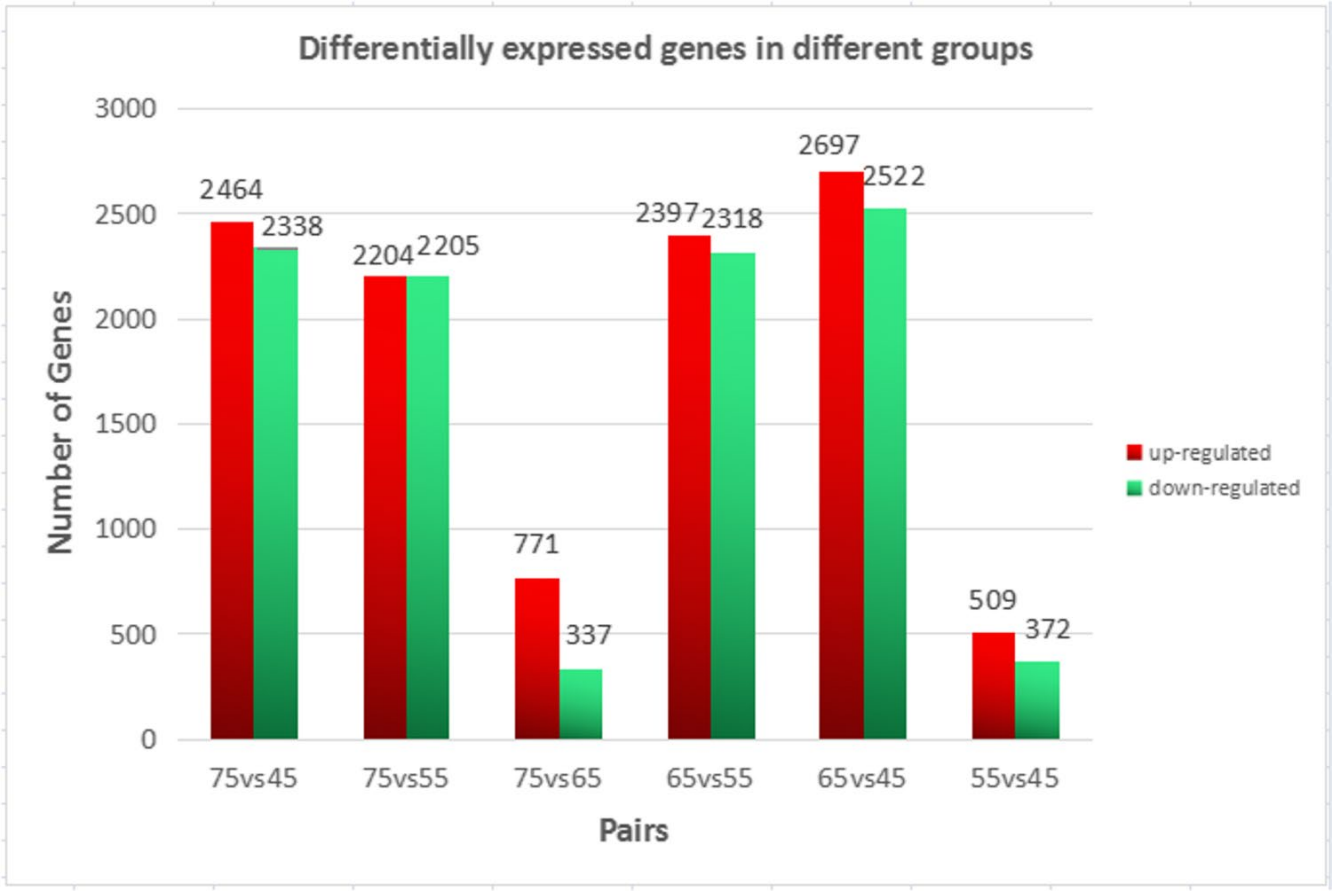
Differentially expression mRNAs in different groups. The red column represents upregulated mRNA and the green column represents downregulated

Inner Mongolia cashmere goats at 4 fetal stages (45d, 55d, 65d, and 75d). Six sets of data were obtained: (d75vsd45, d75vsd55, d75vsd65, d65vsd55, d65vsd45, d55vsd45) (Figs. 2 and 3). The results of miRNAs expression (Fig. 2) showed that there were differences in the number of genes expressed: among the groups d75vsd45 has the largest number of differentially expressed miRNA.There were 327 differentially expressed genes of which 181 were upregulated miRNAs and 146 downregulated miRNAs. It is speculated that the occurrence and development of cashmere follicle will start in the first 65 to 75 days. It is speculated that miRNA is involved in the regulation of the morphogenesis and development of cashmere goat hair follicle.

The results of mRNAs expression (Fig. 3) showed that there were differences among the groups, but the overall upregulation of mRNAs expression was higher than the downregulation of mRNAs. And d65vs45d has the largest number of differentially expressed mRNA. There are upregulated 2697 mRNA and downregulated mRNA 2522, and the number of differentially expressed genes was as high as 5219. Therefore, it is speculated that the formation of hair follicle in cashmere goat is regulated by many genes, mainly positive regulation.

### Validation of differentially expressed miRNAs and mRNAs

**Fig. 4:**
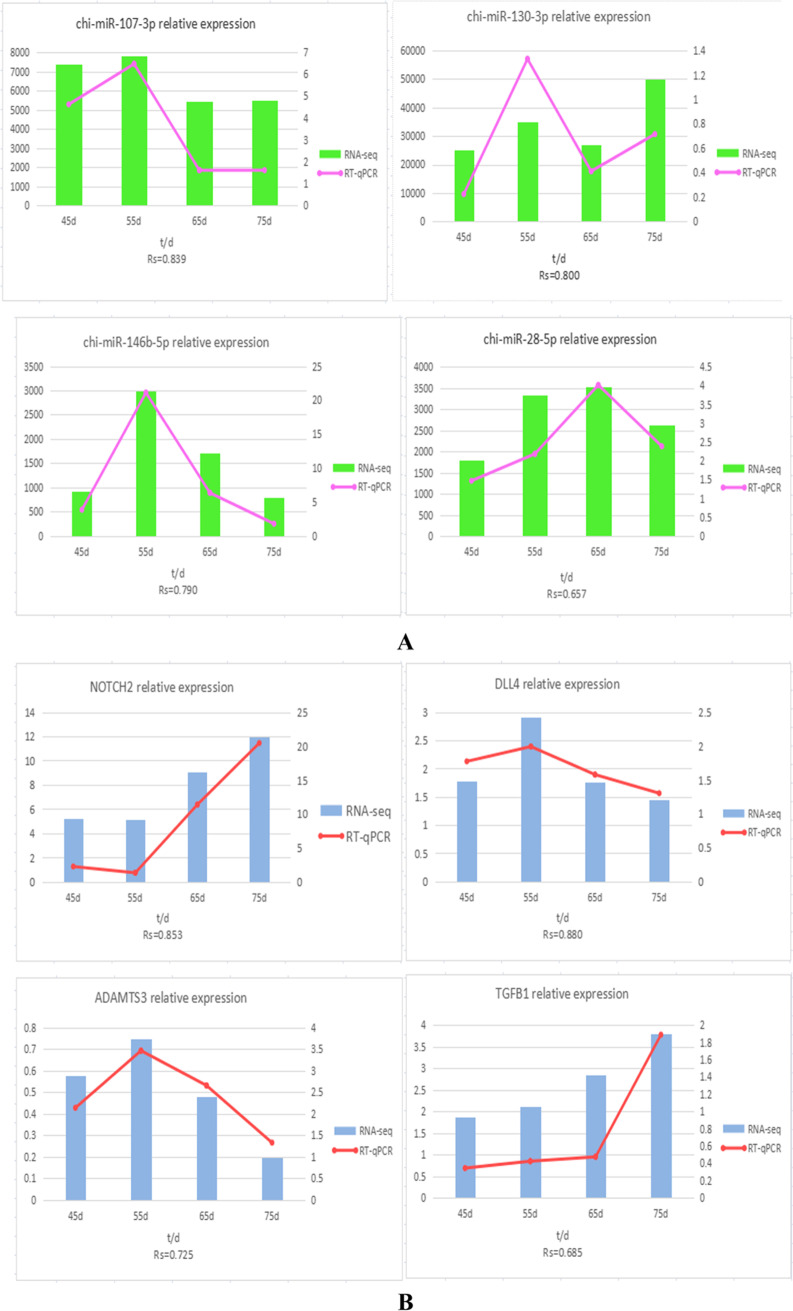
Validation of differentially expressed miRNAs(**A**), mRNAs (**B**) at the 45d, 55d, 65d, and 75d by qRT-PCR

We selected 4 mRNAs and 4 miRNAs which were more studied in the skin follicle for RT-qPCR verification (Fig. 4). All amplification initiates before the 30th cycle. This suggests that the amplification CT values are to be believed, dissolved no bimodal curve, for single piece, explain primers specificity is good. Overall, this analysis can show that the qRT-PCR results are consistent with transcriptome sequencing data.

### Screening of miRNAs and mRNAs related to the morphogenesis and development of secondary hair follicles

**Fig. 5:**
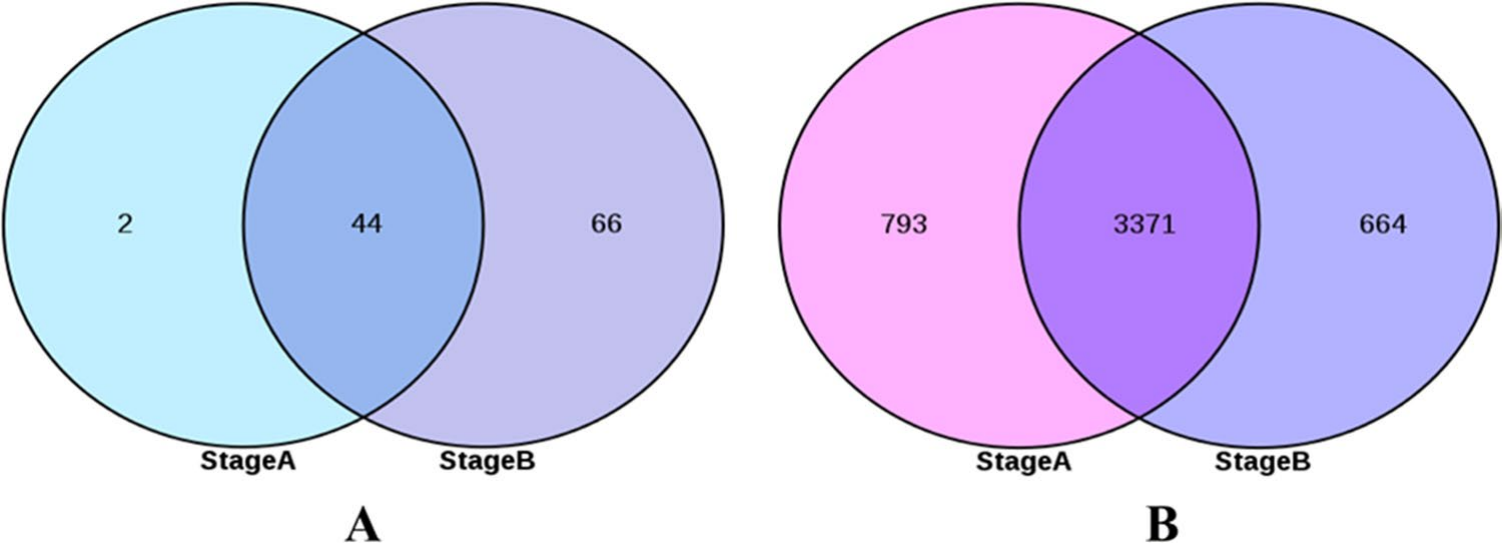
miRNAs (**a**) and mRNAs (**b**) associated with secondary hair follicle morphogenesis and development

Stage A is (65dvs45d, 65dvs55d, and 55dvs45d) differentially expressed miRNA and mRNA, stage B is (75dvs45d, 75dvs55d, 75dvs65d) differentially expressed miRNA and mRNA. It was found that the number of differential miRNAs in stage a was 46 and that in stage B was 110. The 44 differential miRNAs shared by stage B and stage a were screened out, and the remaining 66 miRNAs were used as miRNAs related to secondary hair follicle development (Fig. 5A). At the same time, the number of differential mRNA in stage a was 4164, and that in stage B was 4035. The total number of 3371 differential mRNA in stage B and stage a was screened out, and the remaining 664 mRNA were used as the mRNA related to secondary hair follicle development (Fig. 5B).

### Functional analysis of secondary hair follicle morphogenesis and development related mRNA

**Fig. 6:**
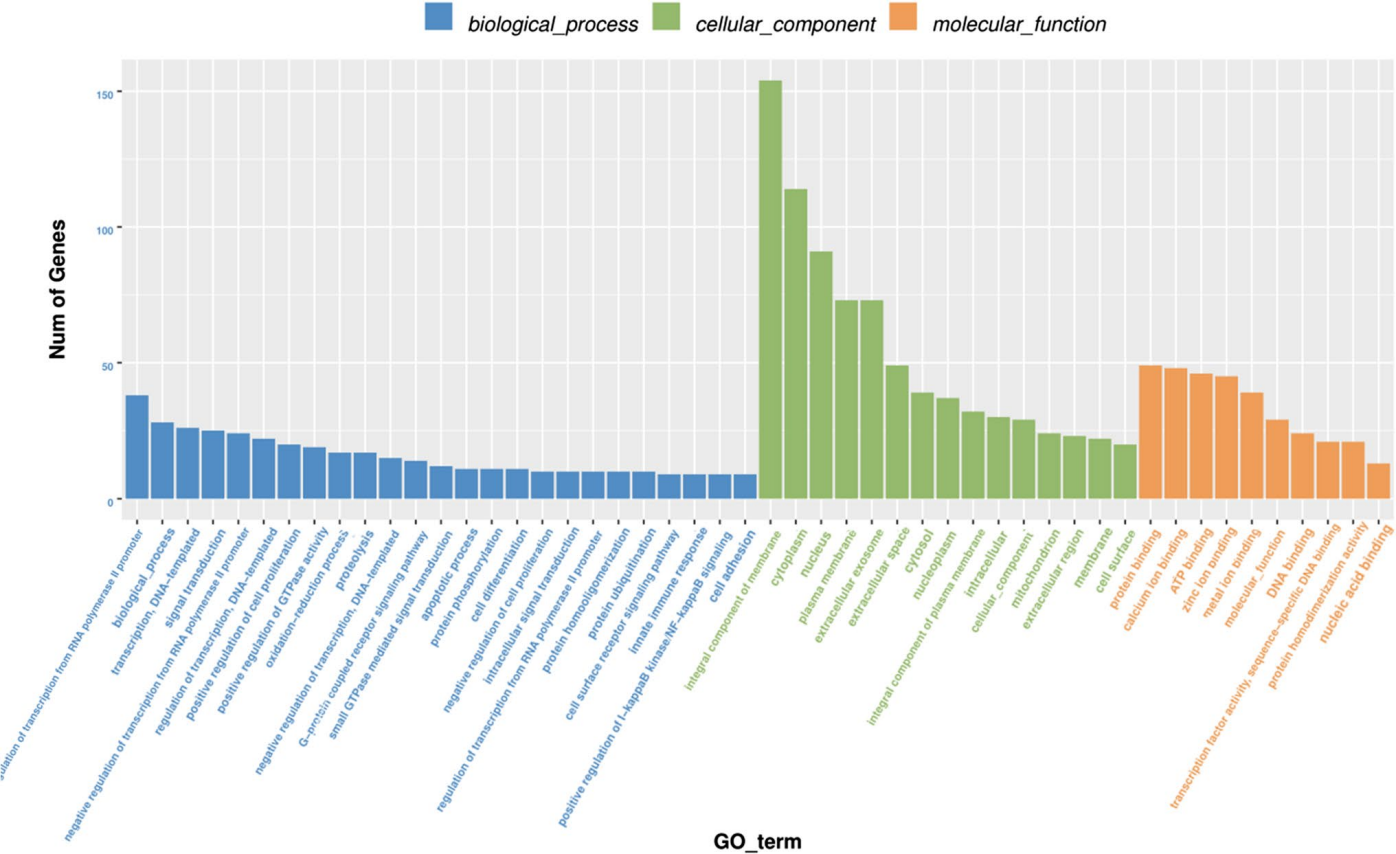
GO analysis of differentially expressed mRNAs related to the morphogenesis and development of secondary hair follicles

The 664 differentially expressed genes related to the morphogenesis and development of secondary hair follicles were screened for GO enrichment analysis and KEGG pathway analysis. The enrichment analysis results showed that the differential genes related to secondary hair follicle development were enriched in 2563 GO team, of which 344 were important enrichments (*p* < 0.05) (Table S1). The results of biological process analysis indicated that these genes related to the morphogenesis and development of secondary hair follicles mainly involved in stem cell differentiation, positive regulation of cell proliferation, and positive regulation of NF-kappaB signaling. Moreover, based on the cellular component analysis, the genes related to the morphogenesis and development of secondary hair follicles were found to be related to cytosol, intracellular, and extracellular space. In addition, results of molecular function analysis showed that the genes related to the morphogenesis and development of secondary hair follicles were associated with ATP binding, DNA binding, and protein binding (Fig. 6).

**Fig. 7:**
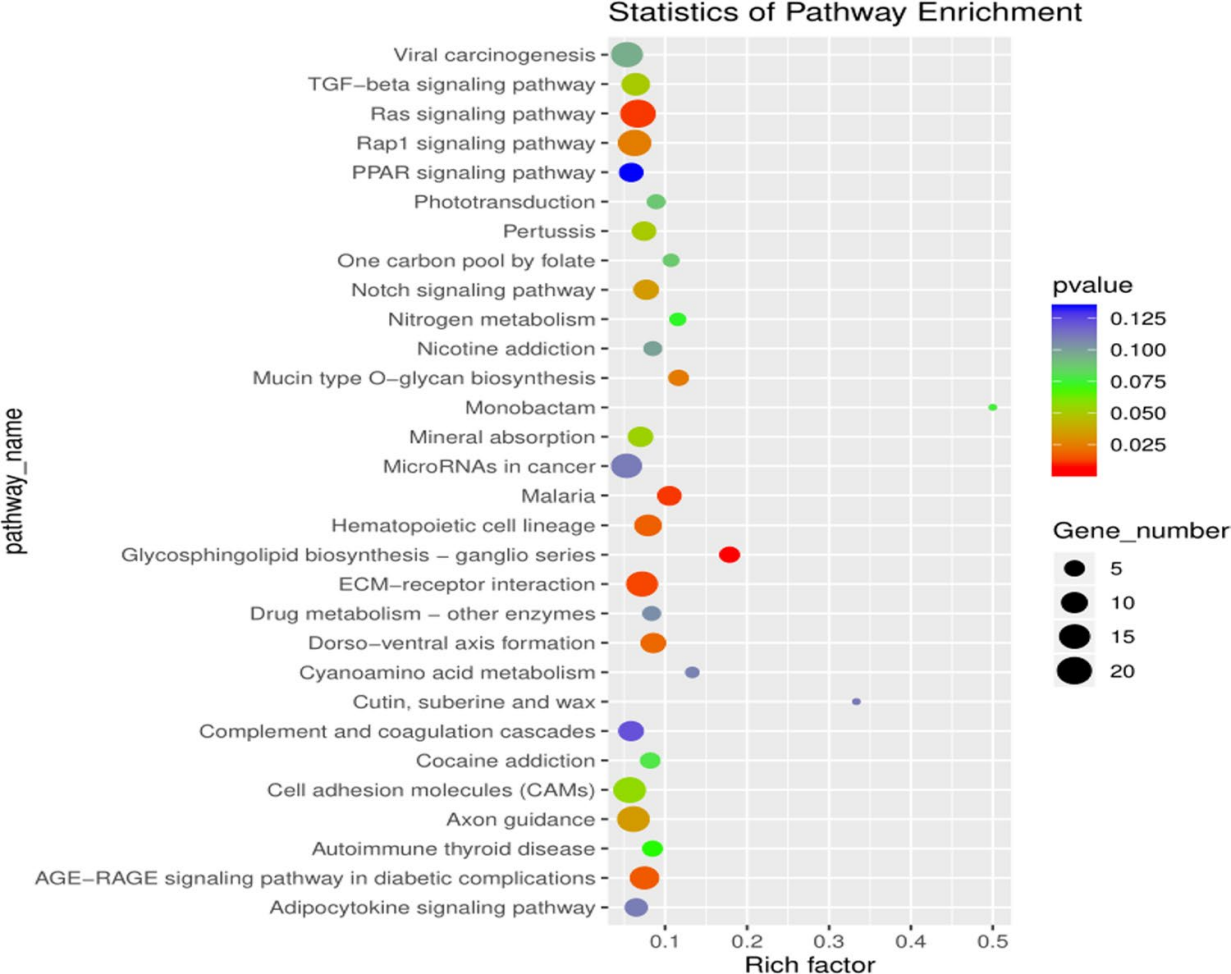
KEGG analysis of differentially expressed mRNAs related to the morphogenesis and development of secondary hair follicles

KEGG pathway analysis showed that a total of 260 pathways were enriched. Of these, the top 20 entries with *P* value are important pathways (TableS2). The results indicated several enriched pathways were related to the morphogenesis and development of hair follicles, such as TGF-beta signaling pathway, Notch signaling pathway, and so on (Fig. 7).

### Combined analysis of miRNA and mRNA

**Fig. 8:**
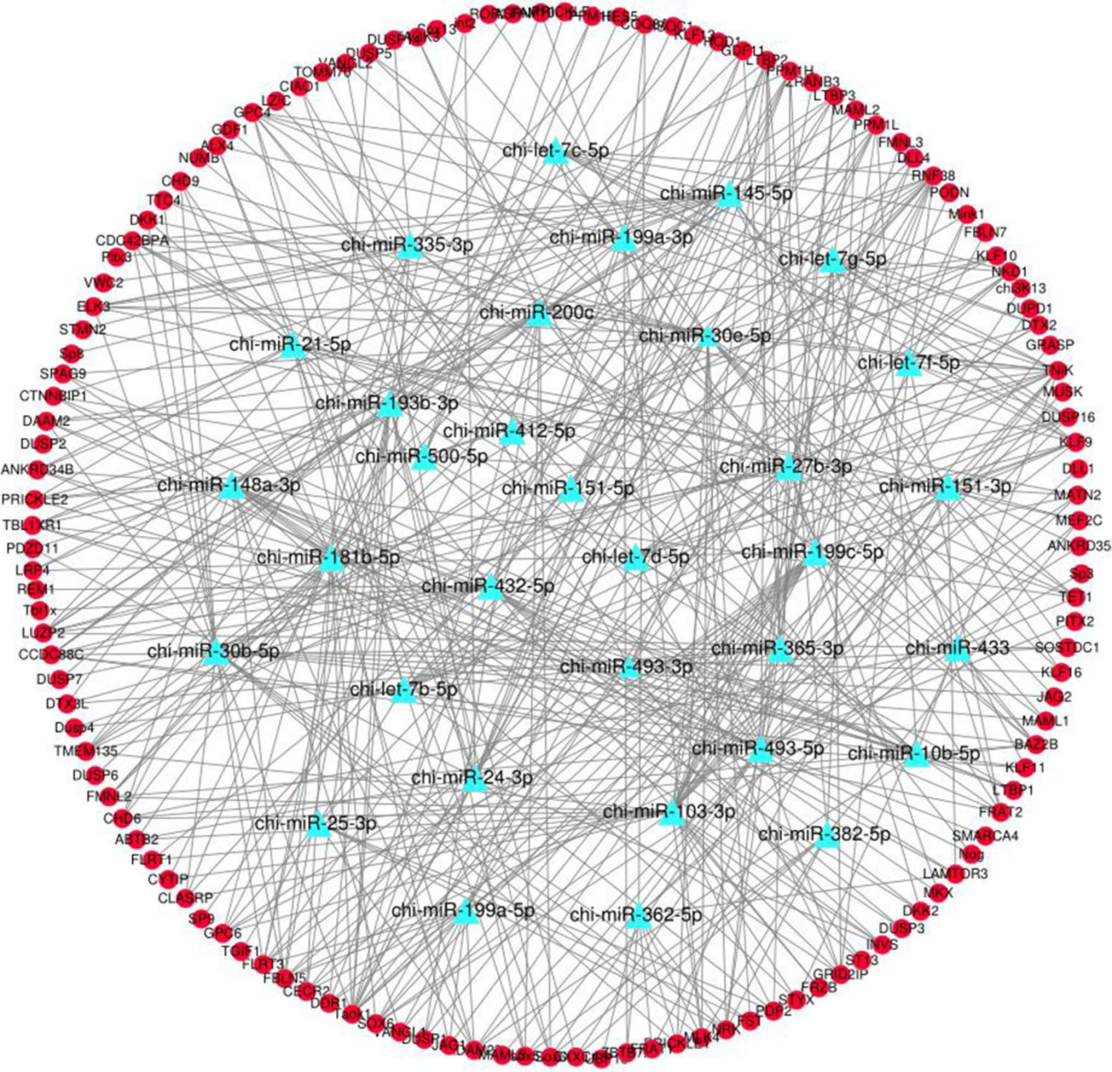
33 miRNAs related to secondary hair follicle development and their target genes

**Fig. 9:**
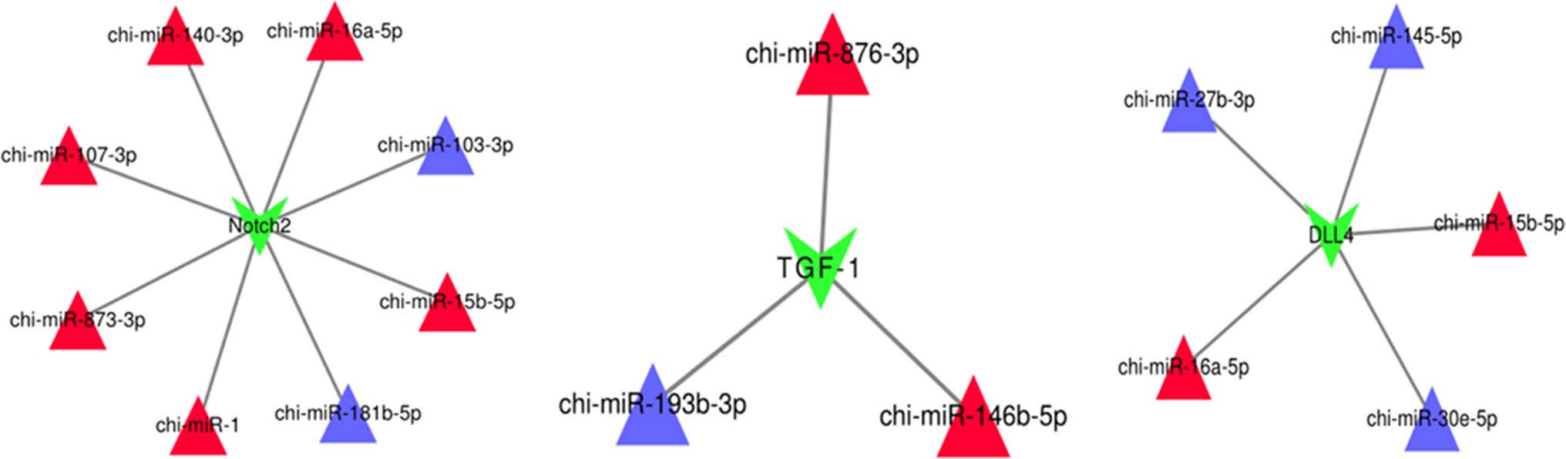
mRNA in important pathways and its targeted miRNA.

Predicted 33 high-expressing miRNAs target genes related to the development of secondary hair follicles (Fig. 8). Constructed miR-145-5p-DLL4, miR-27b-3p-DLL4 and miR-30e-5p-DLL4, miR-193b-3p-TGF-β1, miR-181b-5p-NOTCH2, and miR-103-3p-NOTCH2 regulatory network related to the development of secondary hair follicles (Fig. 9). Used Cytoscape software to visualize the above results.

### Functional analysis of chi-miR-30e-5p and chi-miR-145-5p targets DLL4

**Fig. 10:**
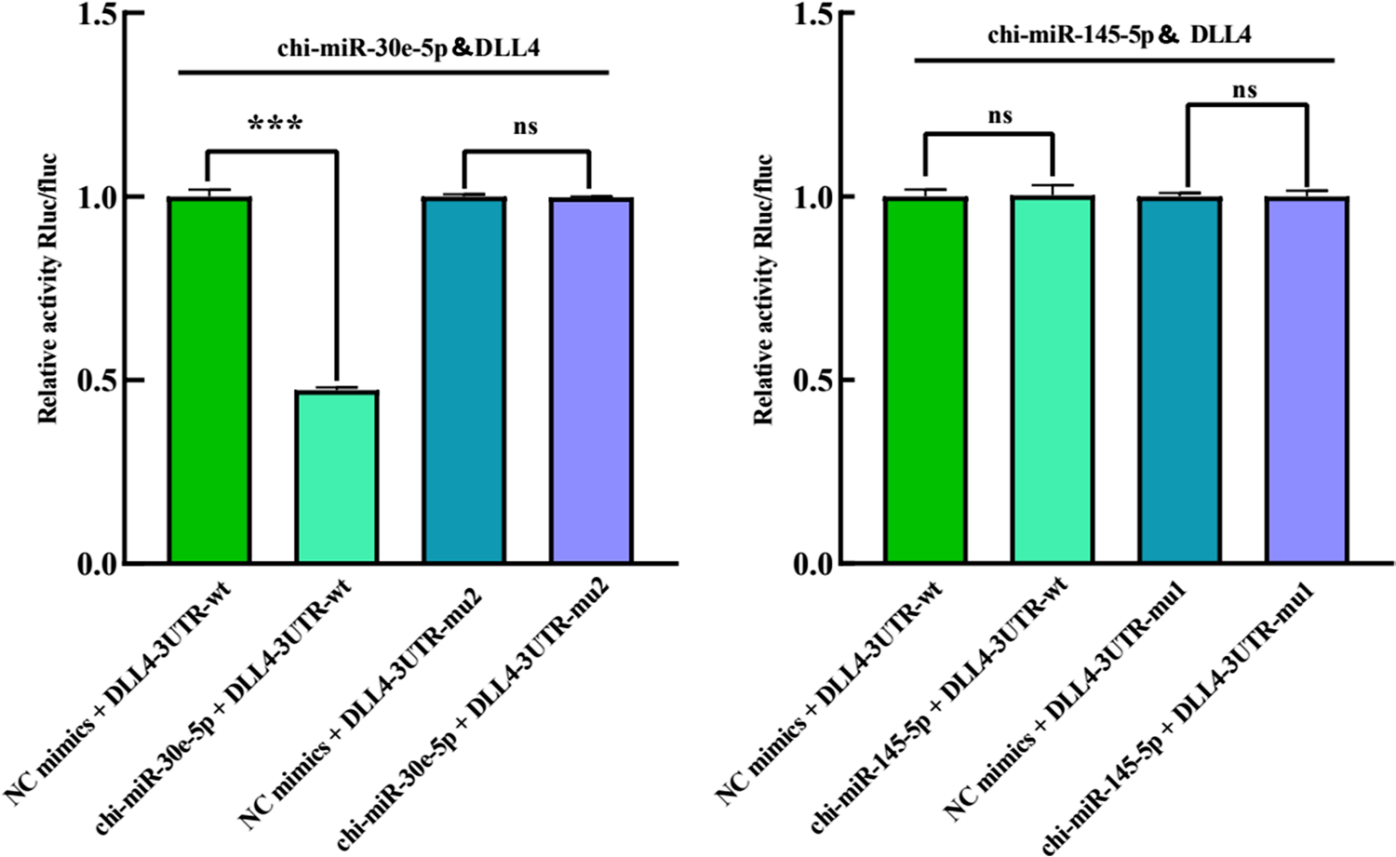
Verification of the targeting relationship between chi-miR-145b-5p,chi-miR-30e-5p and DLL4 in Inner Mongolia cashmere Goat. (**A**) chi-miR-145-5p and DLL4 3’-UTR binding sites and mutation sites. (**B**) chi-miR-30e-5p and DLL4 3’-UTR binding sites and mutation sites. (**C**) Verification of the interaction between chi-miR-30e-5p and DLL4 3’-UTR detected by a dual-luciferase reporter gene assay (*P* > 0.05) (**D**) Verification of the interaction between chi-miR-30e-5p and DLL4 3’-UTR detected by a dual-luciferase reporter gene assay (***P < 0.001). Results in **C** and **D** are expressed as mean 6 standard error of the mean (SEM)

Targetscan and miRanda software predicted that circRNA3236 was targeted to chi-miR-145-5p, chi-miR-145-5p, and chi-miR-27b-3p, and there was a binding site respectively. Therefore, mutant vectors were constructed to verify the specific binding sites (Fig. 10A and B). The results showed that: compared with the NC group, chi-miR-30e-5p significantly decreased the expression of luciferase in DLL4 WT (*p* < 0.001). It shows that there is a binding site between the chi-miR-30e-5p and DLL4. After mu2 mutation, chi-miR-27b-3p failed to down regulate the expression of luciferase in circRNA3236-mut1 (*p* > 0.05), indicating that the mutation was successful. Mu2 is the binding site of chi-miR-30e-5p and DLL4-wt. The results showed that compared with the NC group, chi-miR-145-5p cannot significantly decreased the expression of luciferase in DLL4 WT (*p* > 0.05), indicating that chi-miR-145-5p are not able to targets DLL4 (Fig. 10C and D).

## Discussion

With the development of high-throughput sequencing technology, miRNA research has made great progress from human diseases to other animals. In recent years, there are many studies on the regulatory role of miRNA in hair follicle development cycle, but the regulatory mechanism of miRNA in hair follicle development of cashmere goat skin is relatively scarce. In this study, high-throughput sequencing technology was used to sequence the transcriptome of Inner Mongolia Cashmere Goat at 45, 55, 65 and 75 days of fetal period, and the expression profiles of miRNA and mRNA were obtained. According to the morphological development of fetal hair follicles in different periods, miRNA and mRNA related to secondary hair follicle development were screened. The related mRNA was significantly enriched in Notch, TGF-β, and other important signaling pathways related to hair follicle development. Notch signaling pathway contains a variety of Notch receptors and notch ligands. Notch receptor encoded by Notch gene is a single transmembrane protein containing multiple EGF like repeats and CDC10/Ankyrin repeats. Its carboxyl terminal is in the cytoplasm and its amino terminal is outside the cell. There are four Notch homologous molecules discovered by human beings, namely Notch l, 2, 3, and 4. There were 36 EGF like repeats in Notch 1 from the extracellular amino terminal to the intracellular carboxyl terminal, Notch 2 also contained 36 EGF like repeats, while Notch 3 contained 34 EGF like repeats, Notch 4 contains 29 EGF like repeats (Thomas 2002; Logeat et al. 1998). In Drosophila, Notch ligands are Delta and Serrate. There are three Delta genes (DLLl, DLL3, and DLL4) encoding Delta like, 3 and 4 ligand proteins, respectively. These ligands are single transmembrane glycoproteins, and their extracellular domain contains different numbers of EGF like repeats. There is a DSL motif at the N-terminal that is necessary for binding Notch receptor (Smas and Sul 1993).

Notch signaling is directly related to hair follicle morphogenesis (Lin et al. 2000). Notch signaling pathway may be involved in the early stage of hair follicle development, and play an important role in the late hair stem differentiation. In this study, DLL4, Notch2, and other genes were enriched in Notch signaling pathway. It is found that Notch2 has potential significance in regulating cashmere fineness (Di 2016). DLL4 is related to the development of wool follicle (Zhang 2014). The Notch pathway often functions as an executor. At home and abroad, there are many studies on the genes related to this pathway in human and mouse skin hair follicles. In mouse embryo stage, Notch 1 is mainly expressed in the basal plate of hair follicle, while Delta 1 is expressed in the dermal cells which are about to form dermal papilla. Ectopic expression of Delta1 in a small area of epithelium can promote the expression of Notch 1 and Notch 2 and accelerate the formation of basal plate, at the same time, it can inhibit the formation of basal plate in the cells around the basal plate (Crowe et al.^1998^). After inhibition of Notch1 receptor activity in mouse embryos and postnatal adults, it was found that the hair follicle substrate formed prematurely. After inactivation of Notch1 receptor, the growth period of embryonic hair follicles and adult hair follicles shortened and entered the degenerative stage ahead of time. The hair follicles remained in the resting stage permanently, and no new hair follicles were produced (Lin et al. 2000). Delaro et al. first discovered transforming growth factor (TGF-β) when they studied virus in 1978, and then found some substances with similar functions, which are called TGF-β superfamily (Roberts 1998; Dia 2007). There are five isomers of TGF-β, which are related in structure and similar in sequence. Only three subtypes (TGF-β1, TGF-β2, and TGF-β3) were found in mammals (Atamas and White 2003). At present, it has been found that there are five subtypes of TGF-β receptor, which are TGF-β R1, TGF-β R2, TGF-β R3, TGF-β R4, and TGF-β R5, and now the first three receptors are mainly studied (Massague et al. 2000). In this study, TGF-β1 was enriched in TGF-β signaling pathway. It was found that TGF-β 1 could regulate the hair follicle telogen in mice, and the transformation from anagen to telogen was significantly delayed in TGF-β 1 knockout mice (Foitzik et al. 2000); TGF-β 2 can inhibit the elongation of hair stem and induce the morphological changes of hair follicle telogen (Foitzik et al. 1999); at the same time, it was found that TGF-β 2 may induce apoptosis of hair follicle keratinocytes by regulating endogenous apoptosis, and then inhibit hair growth (Tsuji et al. 2003). At the same time, TGF-β ligand can also regulate hair follicle development under the action of miRNA. Studies have shown that mir-18b inhibits TGF-β 1-induced differentiation of hair follicle stem cells into smooth muscle cells by targeting SMAD2 (Liu et al. 2013). chi-mir-199a-5p regulates the development of cashmere goat hair follicles by targeting the expression of TGF-β 2 (Han et al. 2020).

miRNA is a non-coding RNA that has been studied earlier in skin and hair follicles. In vivo, miRNA binds to the untranslated regions (UTR) at the 3 'end of mRNA to achieve the function of inhibiting mRNA translation protein without reducing the amount of mRNA (Sun et al. 2010). As key regulators of post transcription factors and transcriptional gene network, miRNAs play an important role in the production of hair follicle cells (Kopecky 2011; Marson et al. 2008). In this study, we used targetScan and miRanda software to predict 33 high expression miRNA target genes related to secondary hair follicle development, and initially constructed miRNA mRNA regulatory network. The results of dual-luciferase reporter gene assay indicated that there is a targeted relationship between chi-miR-30e-5p and DLL4.Studies have shown that some miRNAs regulate the development of hair follicles and the periodic growth of villi. The expression of chi-miR-30b-5p was higher in the resting period than in the growing period of white cashmere in Shanbei, and it could inhibit the proliferation of dermal papilla cells by targeting CaMKII δ (Zhang et al. 2020); miRNA-203 may regulate hair follicle development of cashmere goats by targeting DDOST and NAE 1 (Ma et al. 2021); chi-miR-130b-3p regulates hair follicle development of Inner Mongolia cashmere goat embryo skin by targeting Wnt10A (Wu et al. 2021); miR-let-7a regulates the follicle cycle of Liaoning cashmere goat by acting on C-myc and FGF5 (Ma et al. 2018).

## Conclusion

In this study, we established a small RNA library and mRNA expression profile of cashmere goat skin at 45 days, 55 days, 65 days, and 75 days, identified the differentially expressed miRNA and mRNA, screened the miRNA and mRNA related to secondary hair follicle development, and enriched and analyzed the important pathway (TGF beta signaling pathway, Notch signaling pathway) related to hair follicle development. The regulatory relationships of miR-145-5p-DLL4, miR-27b-3p-DLL4, miR-30e-5p-DLL4, miR-193b-3p-TGF-β1, miR-181b-5p-NOTCH2, and miR-103-3p-NOTCH2 related to secondary hair follicle development were constructed, and the results of dual-luciferase reporter gene assay indicated that there is a targeted relationship between chi-miR-30e-5p and DLL4, which laid a foundation for the joint analysis of the regulatory mechanism of miRNA and mRNA in secondary hair follicle development of cashmere goats.

## Supplementary Information

Below is the link to the electronic supplementary material.

Supplementary file1 (XLSX 41 KB)

Supplementary file2 (XLSX 12 KB)

## Data Availability

The RNA-Seq data were submitted to the SRA database under accession number (SRR13306949,SRR13306948,SRR13306947,SRR13306946,SRR13306945,SRR13306944,SRR13306943,SRR13306942,SRR13306941,SRR13306940,SRR13306939,SRR13306938). Additional data can be found in supplementary files.
